# Thermodynamic Quality Control Reshapes Spatially Resolved Metabolic Flux Predictions in Prostate Cancer

**DOI:** 10.64898/2026.06.25.734531

**Published:** 2026-10-21

**Authors:** Mohammad Reza Zargar, Sunayana Malla, Rajib Saha, Ratul Chowdhury

## Abstract

Spatial transcriptomics enables region-resolved investigation of tumor metabolism, yet translating gene expression into reliable metabolic flux predictions remains challenging because genome-scale models can admit thermodynamically infeasible cycles (TICs). Here, we integrated spatial transcriptomic data from twelve annotated regions of a single prostate tumor, spanning normal, inflamed, and Gleason 3+3 and 3+4 tissues, with Human-GEM using an E-Flux framework incorporating defined nutrient constraints, explicit cycle certification, and loopless flux variability analysis. An initial naïve implementation produced TICs in all twelve flux solutions, with total internal fluxes 190–890-fold higher than those obtained using the revised workflow. All flux solutions were certified free of TICs, and 94.7% of 17,592 loopless flux bounds were determined exactly. Within the analyzed tissue, predicted metabolic similarity was associated with spatial region (permutation p = 0.023) but not histological state (p = 0.52), revealing model-predicted heterogeneity even between regions sharing the same Gleason grade. Flux-based target prioritization identified fatty acid uptake-associated genes (CD36, FABPs, and SLC27A1/4), although the dominant uptake signal was confined to one malignant region, suggesting a localized metabolic hypothesis rather than an established therapeutic vulnerability. Analysis of the zinc–citrate–aconitase axis further demonstrated that zinc-dependent citrate redistribution was absent under baseline conditions and emerged only when oxidative demand, elimination of a cytosolic aconitase bypass, and strong mitochondrial aconitase inhibition coincided. These findings establish the importance of thermodynamic quality control and physiologically informed constraints in spatial metabolic modeling, while distinguishing computationally feasible flux predictions from experimentally validated metabolic mechanisms. The resulting framework provides a reproducible foundation for investigating spatial metabolic heterogeneity and prioritizing testable hypotheses for future experimental validation.

